# The benefits of photobiomodulation in animal models of multiple sclerosis: a systematic review and meta-analysis

**DOI:** 10.3389/fneur.2024.1482096

**Published:** 2024-10-22

**Authors:** Zubair Ahmed

**Affiliations:** Neuroscience and Ophthalmology, Department of Inflammation and Ageing, School of Infection, Inflammation and Immunology, College of Medicine and Health, University of Birmingham, Birmingham, United Kingdom

**Keywords:** multiple sclerosis, photobiomodulation, experimental allergic encephalomyelitis, clinical signs, tumor necrosis factor-alpha, interleukin-10

## Abstract

**Background:**

Photobiomodulation (PBM), using red- or near-infrared light, has been used to treat tendinopathies, nerve injuries, osteoarthritis and wounds and evaluated in experimental allergic encephalomyelitis (EAE). To date, only a few studies have been performed in EAE but surprisingly, a few clinical studies in humans have already been performed, despite the paucity of preclinical evidence.

**Objective:**

Therefore, this study systematically reviewed the usefulness of PBM in ameliorating the clinical signs of EAE, a commonly used animal model of multiple sclerosis, and determine if there is enough evidence to warrant human studies.

**Methods:**

PubMed, EMBASE and Web of Science were searched in July 2024 for studies relating to PBM and EAE without any language restrictions. Since only three studies have been published, all studies were included in the systematic review and data related to clinical signs of EAE was pooled together to conduct a meta-analysis. Non-homogenous data was also reported and thematically synthesized.

**Results:**

A meta-analysis of the pooled data from the three included studies demonstrated a significant reduction of the clinical severity of EAE, with a mean reduction of 1.44, 95% CI (−2.45, −0.42), *p* = 0.006. PBM also significantly reduced other parameters such as infiltration of mononuclear cells, CNS demyelination, apoptosis markers and pro-inflammatory cytokines. However, there was an overall high risk of bias in all of the studies.

**Conclusion:**

The meta-analysis supports the use of PBM to ameliorate the symptoms of EAE, but the paucity of studies and the high risk of bias in the included studies warrants further preclinical investigation before conducting human studies.

## 1 Introduction

Multiple sclerosis (MS) is an inflammatory autoimmune neurodegenerative condition of the human central nervous system (CNS) that is caused by Th1 and Th17 lymphocytes, which induce a response to components of the degenerating myelin (1–3). In the CNS, ongoing inflammation, reactive gliosis (response of glial cells to injury or disease) and axonal injury followed by demyelination characterise the disease, with progressive worsening and increasing disability. MS typically occurs in patients between the ages of 20 to 40 years and affects 2:1 women than men (4, 5). The eventual symptoms of MS include visual loss, spasticity, weakness, impaired walking and coordination, ataxia and bladder problems (5, 6). Fatigue, neuropathic pain and cognitive issues are also common in affected patients and usually manifest before a definitive diagnosis of MS. (7, 8)

Low level laser therapy (LLLT) has been used to treat a variety of tissues such as tendons, nerves, skin, bones, muscle and the CNS (9–12). LLLT modulates cellular processes such as cell and tissue death, promotes wound healing, reduces pain, swelling and inflammation (9, 13–15). The effects of LLLT are mainly through photobiomodulation (PBM), where red- or near-infrared light (range 600–1,000 nm) can be absorbed by the main light sensitive chromophore in mitochondria, cytochrome c oxidase (the terminal electron acceptor of the respiratory chain in mitochondria). This leads to cellular respiration, formation of ATP, modulation of oxidative stress and production of nitric oxide (NO) which can trigger cell signalling and gene expression transcription. PBM applied transcranially to the brain, has been shown to regulate a variety of processes including regulation of microglial function through Src kinases (a non-receptor tyrosine kinase that is activated by oxidative events) (16). PBM also promotes neuronal survival and axon regeneration after spinal cord injury (12) and reduces long-term neurological deficits after traumatic brain injury (17).

Experimental allergic encephalomyelitis (EAE) is the most well-established animal model of MS, caused by CD4^+^ T-cell-mediated inflammatory demyelination of the CNS in rodents (18). Much of the understanding of the pathogenesis of MS is based on studies using the EAE model and it is generally accepted that autoreactive, myelin-specific T cells (T cells which migrate across the blood-brain barrier and mediate damage of neurons and their myelin sheaths) initiate the disease and progression occurs through secretion of proinflammatory cytokines that includes interferon-gamma (IFN-γ), interleukin (IL)-17 and tumor necrosis factor alpha (TNF-α) (19). Recovery from EAE and disease amelioration is thought to occur through anti-inflammatory cytokines such as IL-10 and IL-4 (20). There are a just a few studies of the use of PBM in EAE. However, at present, there are no systematic reviews on the efficacy of PBM therapy in EAE that can be used to support further translational studies or progression into clinical trials. Therefore, the aim of this study was to systematically review the data regarding the use of PBM in EAE and determine its benefits in ameliorating the clinical signs of disease.

## 2 Materials and methods

### 2.1 Search strategy

This systematic review and meta-analysis was performed under the preferred reporting of items for systematic reviews and meta-analysis (PRISMA) statement guidelines (21). A comprehensive search was conducted in PubMed, Scopus, EMBASE and Web of Science in July 2024. The following search strategy along with Boolean and MeSH terms were used for the search: (photobiomodulation OR low-level light therapy OR low-level laser therapy OR red-light therapy OR LLLT OR PBM) AND (multiple sclerosis OR MS OR experimental allergic encephalomyelitis OR EAE OR experimental autoimmune encephalomyelitis). Moreover, a manual search of references was used to expand the yield of further relative studies.

### 2.2 Eligibility and study selection

Since there were only three studies evaluating PBM in EAE, all studies were included without language restrictions. Literature reviews, commentaries, conference papers or editorials were all excluded.

### 2.3 Data extraction

A predesigned Excel sheet was used to capture data from animal studies, including information regarding: study characteristics (author, year of publication, country) and study details (e.g., number of animals, EAE induction protocol) as well as PBM treatment regimen. Primary outcomes related to amelioration of clinical scores of EAE by PBM therapy whilst secondary outcomes included data collection on infiltration of mononuclear cells into the CNS, levels of nitric oxide in spinal cords, TUNEL^+^ cell counts and changes in pro- and anti-inflammatory cytokines.

### 2.4 Risk of bias assessment

Risk of bias in included studies was analysed using the SYRCLE risk of bias assessment tool (22), which is specific to animal studies and has been adapted from the Cochrane Collaboration’s tool for assessing risk of bias in RCTs (23), and used by us previously (24–26).

### 2.5 Data synthesis and analysis

A meta-analysis was conducted with pooled data from all three studies related to clinical scores of EAE where homogenous numerical data was available. The meta-analysis was carried out using RevMan version 5.4.1 software from Cochrane Informatics & Technology, using a random effects model and reporting mean differences and 95% confidence intervals. Other data presented in the three studies were heterogenous and hence a thematic, narrative synthesis of all data within each study is presented.

## 3 Results

### 3.1 Study selection

**Figure 1:**
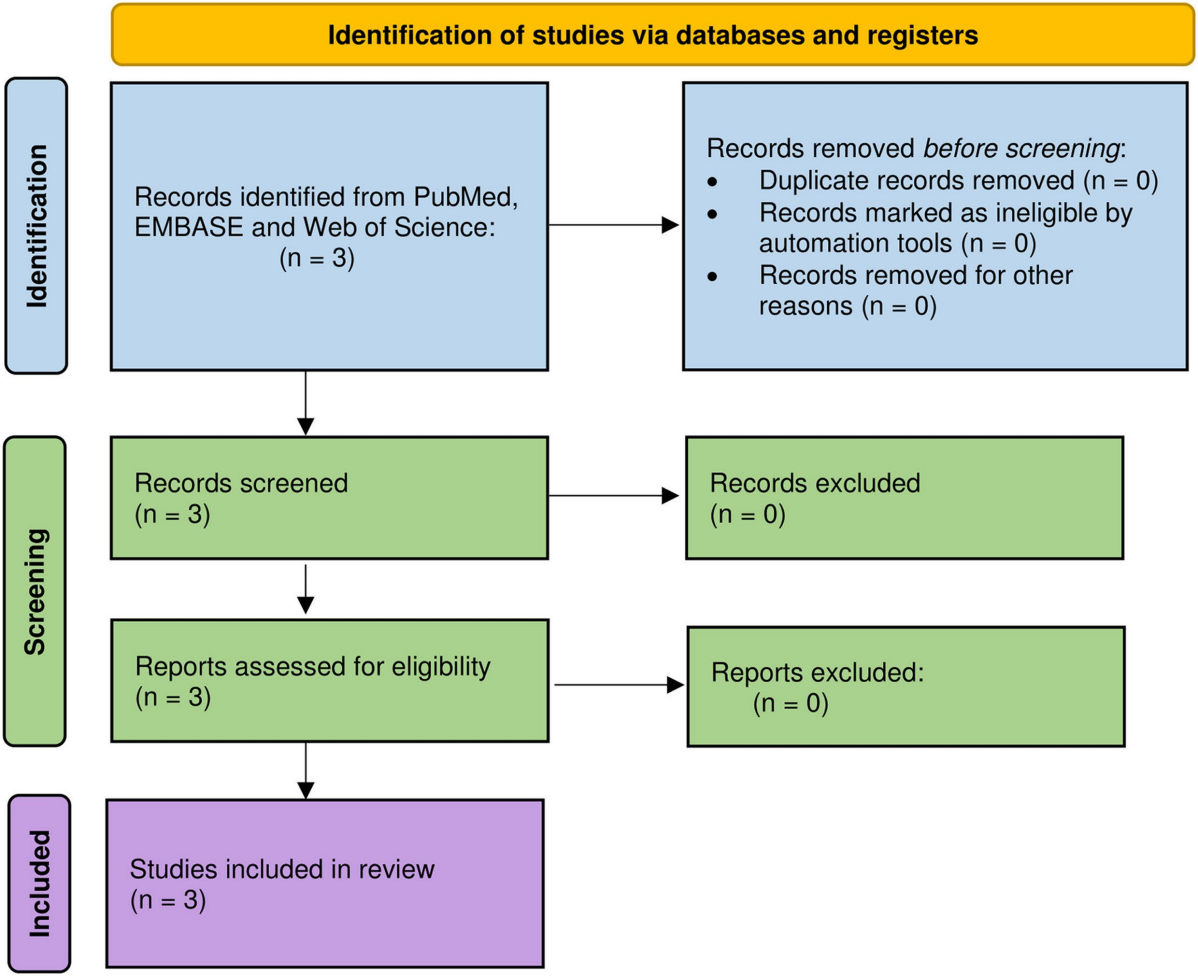
PRISMA flow diagram.

A total of three studies were identified as a result of the comprehensive searches in the three databases as well as searching through the reference list of publications. There were no duplicates to remove, and all three articles were included after the screening process and full-text reading (Figure 1).

### 3.2 Characterization of the included studies

The characteristics of the studies are shown in Table 1. One study was from Brazil (27) whilst the other two studies were from the same lab in the US (28, 29), but all three studies used mouse models of EAE in females. For the EAE induction protocol, one study used MOG_35–55_/complete Freund’s adjuvant (CFA) (27) whilst the other two studies used MOG_35–55_/incomplete Freund’s adjuvant (IFA) followed by either 500 μg or 200 μg of *Mycobacterium tuberculosis*, respectively. All three studies used 660/670 nm PBM wavelength (27–29), whilst one study also used a wavelength of 904 nm (27). There were slight differences in the treatment regimen between the three studies with the two studies from the same lab using the same protocol, including PBM power, fluence and timings (28, 29). For example, PBM parameters in these two studies included a power intensity of 28 mW/cm^2^, energy density of 5 J/cm^2^ and once daily for 3-min exposure times.

**Table 1:** Characteristics of the included studies.

Study	Origin	Induction of EAE	Strain of mouse	*n*	PBM wavelength	PBM treatment parameters	Treatment regime
Goncalves et al. (27)	Brazil	MOG_35–55_/CFA	C57BL/6 (f)	9/group	660 nm &904 nm	660 nm—PBM power of 30 mW, beam area of 0.06 cm^2^, fluency of 10 J/cm^2^ and energy of 0.6 J	20 s exposure for 30 days post-immunisation
						904 nm—PBM power of 70 mW, pulsed for 60 ns, beam area of 0.10 cm^2^, fluency of 3 J/cm^2^	60 nano-second pulses for 30 days post-immunisation
Muili et al. (28)	USA	MOG_35_–_55_/IFA	C57BL/6 (f)	6–8/group	670 nm	PBM power intensity of 28 mW/cm^2^ and energy density of 5 J/cm^2^, once daily for 3 min	Suppression protocol—3 min, once daily for 10 days Onset protocol—3 min, once daily for 7 days Double treatment protocol—3 min, once daily from day clinical signs of diseases onset for 7 days, followed by rest for 7 days and then further 3 min, once daily PBM treatment for 7 days
Muili et al. (29)	USA	MOG_35–55_/IFA	C57BL/6 (f) iNOS^−/−^(f)	6–8/group	670 nm	PBM power intensity of 28 mW/cm^2^ and energy density of 5 J/cm^2^, once daily for 3 min	Suppression protocol—3 min, once daily for 10 days Onset protocol—3 min, once daily for 7 days Double treatment protocol—3 min, once daily from day clinical signs of diseases onset for 7 days, followed by rest for 7 days and then further 3 min, once daily PBM treatment for 7 days

CFA, complete Freund’s adjuvant; IFA, incomplete Freund’s adjuvant; mW, milli watts; J, joules; (f), females.

PBM therapy was delivered by either focussing the laser on the spinal cord at an angle of 90° to the skin, timed to contact at six points on the spinal cord, each 0.5 cm apart (27), or by placing mice in a polypropylene restraint device (12.7 × 9 × 7.6 cm) and PBM delivered using an LED array positioned directly over the animal at a distance of 2 cm, covering the entire chamber and exposing the entire dorsal surface (28, 29). In addition, two studies also employed PBM therapy to several groups of mice in different treatment protocols: suppression protocol (once daily treatment for 10 days starting at 24 h after immunisation), onset protocol (once daily treatment for 7 days starting at the day of onset of clinical signs (score 1.0)) and double treatment protocol (once daily for 7 days on the day of onset of clinical signs (score 1.0)) followed by rest for seven days and then a subsequent seven days of once daily treatments (28, 29).

### 3.3 PBM ameliorates clinical signs of EAE

The clinical signs of EAE were recorded daily using a standard 0–5 scale: 0 = healthy; 1 = loss of tail tone; 2 = hind limb weakness; 3 = paresis or paralysis of one hind limb; 4 = paralysis of both hind limbs and 5 = dead or moribund. PBM therapy significantly ameliorated the severity of EAE in all animals tested, reducing them all back down to scores of 2.2–2.5 (i.e., hind limb weakness/paresis of one hind limb) (Table 2). Use of 904 nm wavelength PBM had the same effect at ameliorating EAE disease severity as 660/670 nm (27).

**Table 2:** PBM therapy ameliorates mean maximum clinical disease score.

Study	Mean maximum clinical score	
	Control	EAE
Goncalves et al. (27)	3.5 ± 0.5	660 nm—2.5 ± 0.5 904 nm—2.5 ± 0.5
Muili et al. (28)	3.9 ± 0.2 NR 4.6 ± 0.2	Suppression protocol—3.6 ± 0.2 Onset protocol—not reported Double treatment protocol—2.2 ± 0.4
Muili et al. (29)	4.5 ± 0.1	3.6 ± 0.3

NR, not reported.

**Figure 2:**
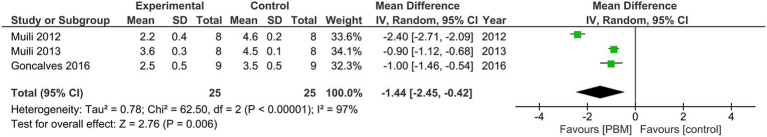
Meta-analysis of the pooled data from the three studies to show significant effects of PBM therapy on clinical score of EAE.

A meta-analysis of the pooled data from all three studies demonstrated that amelioration of EAE clinical scores by PBM therapy was significant, with a mean difference of −1.44, 95% CI (−2.45, −0.42), *p* = 0.006 (Figure 2).

### 3.4 Other effects of PBM therapy in EAE

PBM therapy significantly reduced the levels of nitric oxide (NO) in the spinal cord and spleen, infiltration of mononuclear cells into the CNS and CNS demyelination without affecting lipid peroxidation in EAE treated mice (27) (Table 3). PBM also significantly reduced Bcl-2 and Bax gene expression as well as TUNEL^+^ cells during peak disease and after double treatment of EAE and reduced pro-inflammatory cytokines (IFN-γ and TNF-α) and increased anti-inflammatory cytokines (IL-4 and IL-10) (28, 29) (Table 3).

**Table 3:** Other effects of PBM therapy in the included studies.

Study	Effects of PBM in EAE-induced mice
Goncalves et al. (27)	Significantly reduced NO levels in spinal cord and spleen No change in lipid peroxidation Significantly reduced infiltration of mononuclear cells into CNS Significantly reduced CNS demyelination
Muili et al. (28)	Significantly reduced proinflammatory cytokines IFN-γ and TNF-α Significantly increased anti-inflammatory cytokines IL-4 and IL-10
Muili et al. (29)	Significantly increased Bcl-2and Bax gene expression during peak disease and 2 days after 2nd treatment Significantly decreased TUNEL^+^ cells 2 days post disease onset, during peak disease and 2 days after 2nd treatment

NO, nitric oxide; IFN-γ, interferon-γ; TNF-α, tumor necrosis factor-α; IL-4, interleukin-4.

### 3.5 Risk of bias in included studies

**Figure 3:**
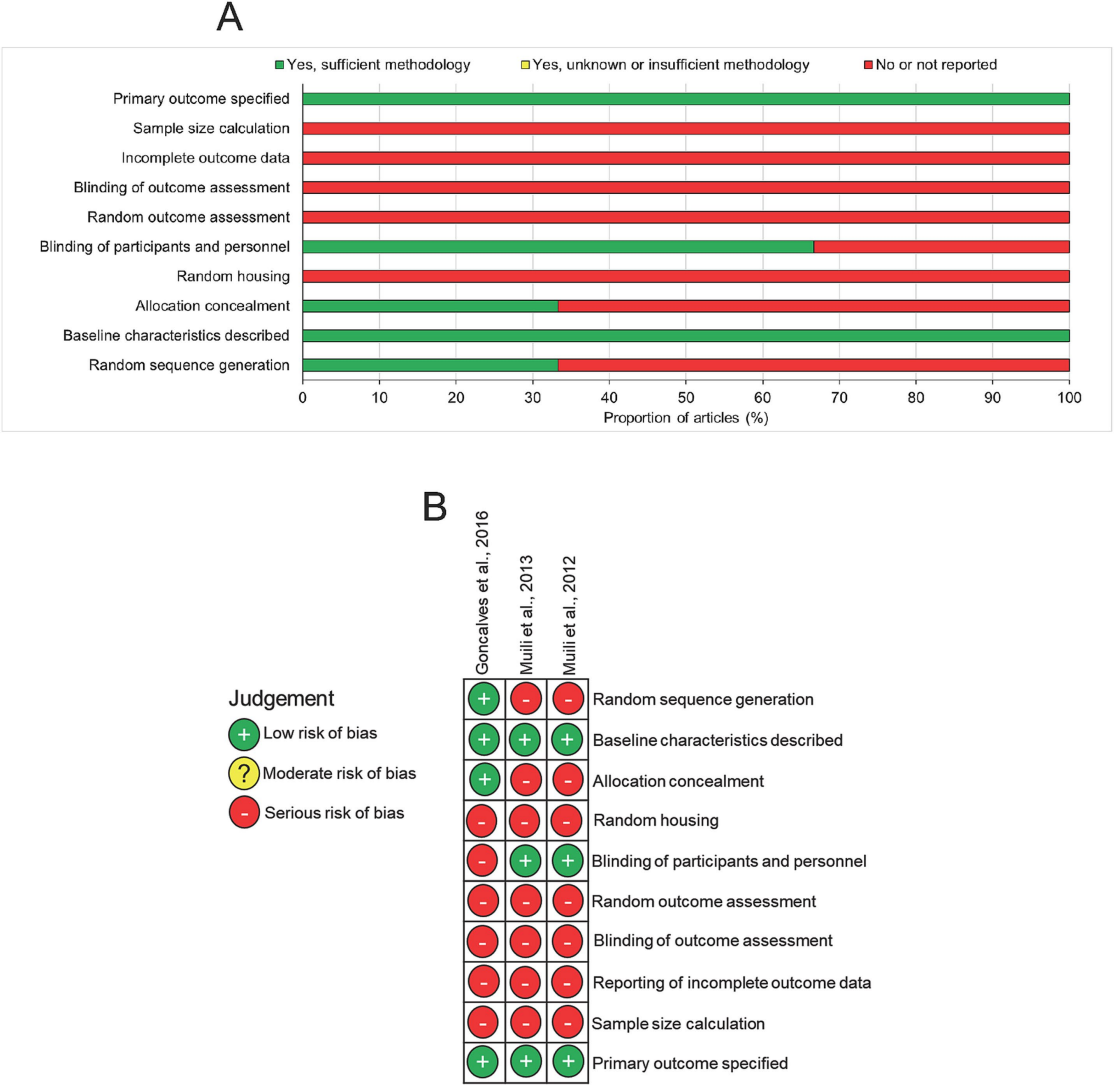
Risk of bias assessment in included studies. **(A)** Summary diagram and **(B)** risk of bias in individual studies.

Analysis of the risk of bias in the included studies using the SYRCLE tool for animal studies showed evidence of high risk of bias in all three studies such that domains including sample size calculation, reporting of incomplete data, blinding of outcome assessment and random outcome assessment were all judged as “no or not reported” (Figures 3A,B). Two of the three studies blinded the participants and personnel and only one study concealed the treatment group allocation and generated a random sequence to treat. However, all three studies specified the primary outcome and described baseline characteristics (Figures 3A,B). In summary, there was an overall high risk of bias in all of the studies.

## 4 Discussion

This systematic search in three databases found only three published studies, which evaluated the effects of PBM in the progression of EAE. All of the studies were performed in female mice using similar EAE induction protocols. We found that PBM can ameliorate the clinical signs of EAE, reducing clinical scores back down to 2.2–2.5. Meta-analysis showed that this reduction in clinical scores was significant. PBM also suppressed NO and infiltration of mononuclear cells into the CNS, reduced markers of cell death and reduced pro-inflammatory cytokines whilst increased anti-inflammatory cytokines. However, the overall risk of bias was judged as high since many of the domains assessed did not report adherence to robust experimental design.

To our knowledge this is the first systematic review and meta-analysis of the effects of PBM therapy on EAE. The fact that we could only find three studies in EAE demonstrates how relatively unexplored this area of research is, given that the first studies in EAE were conducted over a decade ago. There has also been no other reported study in the EAE model since 2016 and hence the use of PBM therapy remains a largely unexplored area of research in EAE. This paucity of studies in the EAE model limits the usefulness of this systematic review but highlights the need for further studies looking into this area of research. We also found another study of the effects of PBM, but in a cuprizone model of demyelination where six sessions of transcranial PBM were delivered to cuprizone-treated mice on three consecutive days, during the third and fourth weeks using 36 J/cm^2^, 50 mW and 0.028 cm^2^ spot area (30). The study found that PBM-treated mice presented with improved motor performance, attenuated demyelination, increased number of oligodendrocyte precursor cells, reduced microglial and astrocyte activation and a milder toxicity to cuprizone (30).

We are unsure why this work has not been followed up by more studies, given the initial promising results in all three studies and the relatively safe, efficacious and non-invasive way of delivering PBM in animal models. The significant reduction of clinical scores of EAE by all three studies points to a potential benefit of PBM in MS as well as reductions in NO in the spinal cord and spleen, which are normally enhanced in MS (31) as well as reduced demyelination, reduced cell death and reduction in pro-inflammatory cytokines and increases in anti-inflammatory cytokines all point to the benefits of PBM to multiple signalling pathways. A significant worry of all the animal studies in our systematic review is the high risk of bias. Whether this is from lack of reporting by authors or a failure to give importance to these parameters in animal studies remains to be determined. However, the high risk of bias in all included studies, especially in domains such as sample size calculation, blinding of outcome assessment, random outcome assessment and reporting of how incomplete outcome data, if any, was handled, means that conclusions from this systematic review must be made with caution. The high risk of bias in animal studies can be avoided by adhering to the standardised techniques in animal experiments based on the Animal Research Reporting of *In Vivo* Experiments (ARRIVE) guidelines (32, 33).

In support of PBM, two studies from the same authors showed benefits of PBM in suppressing IFN-γ and increasing IL-10 production by MS patient-derived peripheral blood mononuclear cells (PBMCs) and CD4^+^ T cells as well as reduced levels of nitrite that correlated with increased production of IL-10 and reduced production of IFN-γ (34). In a randomised controlled trial involving 14 patients with relapsing-remitting MS, PBM therapy to the sublingual region or over the radial artery using 808 nm wavelength and output power of 100 mW for 360 s, twice weekly for 24 sessions, led to significant increases in levels of anti-inflammatory cytokine, IL-10, in peripheral blood from both treatment groups but the levels of nitrites, which are metabolites of nitric oxide and are released by microglia and contribute to oxidative stress and demyelination, were not modulated in either treatment group (35). In two studies on the effects of PBM on muscle function in MS patients: one study shows that PBM treatment had no significant benefits on fatigue, one of the main symptoms of MS which has a negative impact on quality of life and few treatment options (36); whilst another study showed PBM therapy, administered to the belly of the tibialis anterior muscle, improved muscle force recovery and muscle strength in individuals with mild–moderate MS. (37) These studies suggest that there are benefits of PBM in MS patients and further high-quality studies are required to explore these benefits.

One significant advantage of PBM is that it can be delivered non-invasively and shown to have positive benefits. For example, in the EAE studies, PBM was delivered transcutaneously at several spots focussed on the spinal cord or over whole dorsal surface (27–29). In humans with MS, PBM has been delivered sublingually, over the radial artery or to the belly of the tibialis anterior muscle, all non-invasive methods of PBM delivery. There is significant interest in PBM dosimetry since inconsistencies in clinical outcomes of PBM are mainly due to problems in reporting PBM dosing and delivery (38). Therefore, for greater efficacy, other PBM delivery methods may need to be investigated, including implantable PBM devices (12). There is, however, a precedence to use PBM since it is already approved in the UK by the National Institute of Clinical Excellence (NICE) for use in the prevention or treatment of oral mucositis (39).

### 4.1 Limitations

The main limitation of this systematic review was the paucity of studies evaluating the efficacy of PBM in EAE. We found only three studies in animal models of MS that used PBM to monitor benefits in EAE models. Of these three studies, two were from the same lab and hence is an additional limitation. Another limitation is that the treatment regimen was different between studies with the two studies from the same laboratory reporting the same treatment regimen whilst the third study used a different treatment regimen. Finally, the high risk of bias in the three studies means that definitive conclusions from these studies on the usefulness of PBM in EAE models cannot be reliably made.

## 5 Conclusion

This systematic review shows that PBM can significantly reduce clinical signs of EAE in mice with associated benefits in terms of suppressing pro- and increasing anti-inflammatory cytokines, along with reduced demyelination, NO levels and markers of apoptosis. However, the study is limited in only three studies and the high risk of bias makes it difficult to make definitive conclusions about PBM and its usefulness in EAE. There is some additional supporting evidence from a limited number of human studies in MS patients, beginning to reveal some positive benefits to MS patients. These show that PBM not only suppresses pro-inflammatory cytokines and increases anti-inflammatory cytokines, which may help to counter ongoing disease, but also reduces neuron and axon damaging nitrites. To realise the full benefits of PBM however, further studies in the EAE model and more rigorous studies in MS are warranted.
